# Development of a Novel *Escherichia coli–Kocuria* Shuttle Vector Using the Cryptic pKPAL3 Plasmid from *K. palustris* IPUFS-1 and Its Utilization in Producing Enantiopure (*S*)-Styrene Oxide

**DOI:** 10.3389/fmicb.2017.02313

**Published:** 2017-11-27

**Authors:** Hiroshi Toda, Nobuya Itoh

**Affiliations:** Department of Biotechnology, Biotechnology Research Center, Toyama Prefectural University, Imizu, Japan

**Keywords:** *Kocuria rhizophila*, *Kocuria palustris*, styrene monooxygenase, styrene oxide, biphasic reaction system, organic solvent-tolerant biocatalyst

## Abstract

The novel cryptic pKPAL3 plasmid was isolated from the Gram-positive microorganism *Kocuria palustris* IPUFS-1 and characterized in detail. pKPAL3 is a circular plasmid that is 4,443 bp in length. Open reading frame (ORF) and homology search analyses indicated that pKPAL3 possesses four ORFs; however, there were no replication protein coding genes predicted in the plasmid. Instead, there were two nucleotide sequence regions that showed significant identities with untranslated regions of *K. rhizophila* DC2201 (NBRC 103217) genomic sequences, and these sequences were essential for autonomous replication of pKPAL3 in *Kocuria* cells. Based on these findings, we constructed the novel *Escherichia coli*–*Kocuria* shuttle vectors pKITE301 (kanamycin resistant) and pKITE303 (thiostrepton resistant) from pKPAL3. The copy numbers of the constructed shuttle vectors were estimated to be 20 per cell, and they exhibited low segregation stability in *Kocuria* transformant cells in the absence of antibiotics. Moreover, constructed vectors showed compatibility with the other *K. rhizophila* shuttle vector pKITE103. We successfully expressed multiple heterologous genes, including the styrene monooxygenase gene from *Rhodococcus* sp. ST-10 (*rhsmo*) and alcohol dehydrogenase gene from *Leifsonia* sp. S749 (*lsadh*), in *K*. *rhizophila* DC2201 using the pKITE301P and pKITE103P vectors under the control of the glyceraldehyde 3-phosphate dehydrogenase (*gapdh*) promotor. The RhSMO–LSADH co-expressing *K. rhizophila* was used as a biocatalyst in an organic solvent–water biphasic reaction system to efficiently convert styrene into (*S*)-styrene oxide with 99% ee in the presence of 2-propanol as a hydrogen donor. The product concentration of the reaction in the organic solvent reached 235 mM after 30 h under optimum conditions. Thus, we demonstrated that this novel shuttle vector is useful for developing biocatalysts based on organic solvent-tolerant *Kocuria* cells.

## Introduction

Enantiopure epoxides are useful building blocks for synthesizing various chiral materials, including pharmaceuticals, agrochemicals, and fine chemicals (Farina et al., 2006; Patel, 2008). Direct enantioselective epoxidation of prochiral alkenes is a straightforward strategy for producing chiral epoxides, and many chemical approaches have been developed to achieve this objective (Shi, 2004). Sharpless epoxidation is an efficient procedure for oxidizing allylic alcohols using a titanium/tartrate/*tert*-butyl hydroperoxide system to yield corresponding chiral epoxides. Many chemical catalysts that contain transition metals, such as metal–salen complexes and chiral metalloporphyrins, have been developed for the enantioselective epoxidation of alkenes (Katsuki and Sharpless, 1980; Hanson and Sharpless, 1986; Irie et al., 1991; Chang et al., 1994; Tu et al., 1996). Furthermore, the biological synthesis of epoxides using monooxygenases has also been studied (Panke et al., 1998; Martinez and Stewart, 2000; Hollmann et al., 2003; Farinas et al., 2004; Champreda et al., 2006). Biological synthesis of epoxides has several advantages over chemical synthesis, including superior chemoselectivity, regioselectivity, and enantioselectivity, as well as improved environmental sustainability.

The genomes of several styrene-degrading microorganisms contain styrene monooxygenase (SMO) genes, which are involved in the first step of styrene degradation and allow them to catalyze the epoxidation of styrene to enantiopure (*S*)-styrene oxide (Hartmans et al., 1990; Beltrametti et al., 1997; Panke et al., 1998). SMOs consist of two enzymes: flavin adenine dinucleotide (FAD)-dependent monooxygenase (StyA) and NAD(H)-dependent flavin oxidoreductase (StyB). SMOs convert styrene to (*S*)-styrene oxide with high enantioselectivity using NADH as an electron donor (Panke et al., 1999; Hollmann et al., 2003). Many SMOs are well characterized, and their enzymatic properties and substrate specificities have been investigated (Marconi et al., 1996; Panke et al., 1998; Velasco et al., 1998; Lin et al., 2010; Tischler et al., 2012). We have also reported the isolation and characterization of SMO genes from *Rhodococcus* sp. ST-10(RhSMO; Toda and Itoh, 2012; Toda et al., 2012a) and the development of biocatalysis reactions for producing enantiopure epoxides from various aryl- and aliphatic alkenes using RhSMO (Toda et al., 2012b, 2014).

*Escherichia coli* is the microorganism most frequently used as biocatalyst host cells owing to its ease of use and abundant molecular tools for genetic manipulation. However, *E. coli* cells are unsuitable for long-term biocatalysis reactions using organic solvents and toxic compounds because they are easily inactivated by these substances. To overcome this problem, organic solvent-tolerant microorganisms, especially *Pseudomonas putida*, have been assessed as host cells for biocatalysis (Heipieper et al., 2007; Park et al., 2007; Verhoef et al., 2009; Siriphongphaew et al., 2012; Nikel et al., 2014). Recently, we also reported the construction of a biocatalysis system possessing RhSMO that uses the organic solvent-tolerant microorganism *K. rhizophila* DC2201 as a host cell and demonstrated the bioproduction of various enantiopure (*S*)-epoxyalkanes in an organic solvent–water biphasic reaction (Toda et al., 2015). *K. rhizophila* DC2201 is a Gram-positive microorganism belonging to the family *Micrococcaceae* in the order *Actinomycetales*, and its whole genomic DNA sequence was previously determined (Takarada et al., 2008). This microorganism has several advantages for utilization as a biocatalysis host cell, including its organic solvent tolerance, high halotolerance, robust cell structure, and small genome size. Furthermore, it was reported that several *Kocuria* species produce important natural pigments, including astaxanthin and β-carotene, which are widely used as food additives and health supplements (Goodwin, 1980; Porter and Spurgeon, 1981; Dufosse, 2006). It is expected that development of genetic modification tools for these bacteria may contribute to the efficient production of various useful materials such as pharmaceutical and functional food components.

Thus, *Kocuria* species are expected to be useful host cells for biocatalysis. However, only a few genetic tools are available for *Kocuria* cells (Matsumura et al., 2012; Ohta et al., 2014) and little is known about gene transcription, protein expression, and metabolite flux in *Kocuria* cells. Such tools and information are important for increasing the potential uses of host cells by controlling recombinant protein expression and modification of the metabolite pathways of host cells. Therefore, we aimed to both construct genetic tools for use in the genus *Kocuria* and apply them to a biocatalysis system for producing a wide variety of organic compounds.

In our previous study, we reported the isolation and characterization of the two cryptic plasmids pKPAL1 and pKPAL2 from *K. palustris* IPUFS-1 and the construction of the *E. coli*–*Kocuria* shuttle vector pKITE101 series based on pKPAL1 (Toda et al., 2017). These plasmids were inferred to be theta-replicating plasmids with copy numbers of 60 per chromosome in *Kocuria* cells. The constructed shuttle vector was stably maintained in *K. rhizophila* DC2201 cells, and it was available for heterologous gene expression under control of the *gapdh* promoter.

In this study, we isolated and characterized pKPAL3, another novel cryptic plasmid from *K. palustris* IPUFS-1. The whole nucleotide sequence of pKPAL3 was determined, and four putative open reading frames (ORFs) and two regions homologous to *K. rhizophila* DC2201 genomic DNA sequences were confirmed. The copy numbers of *E. coli*–*Kocuria* shuttle vectors pKITE301 and pKITE303 were assayed, and their segregation stability and compatibility with other *E. coli*–*Kocuria* shuttle vectors were examined. Moreover, we constructed a biocatalyst co-expressing RhSMO and LSADH (alcohol dehydrogenase from *Leifsonia* sp. S749) using pKITE301 and pKITE103, and the bioproduction of enantiopure (*S*)-styrene oxide from styrene in an organic solvent–water biphasic reaction system using 2-propanol as a hydrogen donor was demonstrated.

## Materials and Methods

### Chemicals

Styrene and styrene oxide were purchased from Nacalai Tesque, Inc. (Kyoto, Japan). Bis-(2-ethylhexyl) phthalate (DEHP) and other chemicals were purchased from Wako Pure Chemical Industries (Osaka, Japan).

### Culture Strains and Vectors

*Kocuria palustris* IPUFS-1 (Toda et al., 2017) was used as a source of cryptic plasmids. *K. rhizophila* DC2201 (NBRC 103217), *K. kristinae* NBRC 15354, *K. varians* NBRC15358, *K. palustris* NBRC 16318, *K. rhizophila* NBRC 16319, *K. polaris* NBRC 103063, *K. flava* HO-9041 (NBRC 107626), and *K. turfanensis* HO-9042 (NBRC 107627) were used as transformation hosts for the constructed shuttle vectors, while *E*. *coli* JM109 and EC100D *pir*-116 were used for cloning. pHSG298 and pUC118 were used to construct the *E. coli*–*Kocuria* shuttle vectors. pGEM-T Easy Vector (Promega Corp., Fitchburg, WI, United States) was used to construct control plasmids for quantitative polymerase chain reaction (qPCR). The bacterial strains and plasmids used in this study are listed in **Table 1**.

**Table 1:** Bacterial strains and plasmids in this study.

Bacterial strain/Plasmid	Description	Source
**Bacterial strain**		
*Escherichia coli* JM109	F′[*traD36, proAB*^+^*, lac Iq, lacZ*ΔM15]/Δ(*lac-proAB*) *recA*1*, relA*1*, endA*1, *gyrA*96*, thi-*1*, hsdR*17(*r*_K_^-^ *m*_K_^+^), e14^-^ (*mcrA*^-^)*, supE*44,	TaKaRa
*E. coli* EC100D *pir*-116	F^-^ *mcrA* Δ(*mrr-*hsdRMS*-mcrBC*) Δ80d*lacZ*ΔM15 Δ*lac*X74 *recA*1 *endA*1 *araD*139 Δ(*ara, leu*)7697 *galU galK* aaa*-rpsL nupG pir*-116(DHFR)	AR Brown
*Kocuria palustris* IPUFS-1	Wild-type	Toda et al., 2017
*K. rhizophila* DC2201	Wild-type	NBRC
**Plasmid**		
pHSG298	*E. coli* cloning vector	
pUC118	*E. coli* cloning vector	
pKPAL3	4.4 kbp wild-type plasmid from *K. palustris* IPUFS-1	This study
pKES3300	*E. coli*–*Kocuria* shuttle vector harboring 4.4 kbp fragment from pKPAL3; Km^r^	This study
pKES3301	*E. coli*–*Kocuria* shuttle vector harboring 3.4 kbp fragment from pKPAL3; Km^r^	This study
pKES3302	*E. coli*–*Kocuria* shuttle vector harboring 2.4 kbp fragment from pKPAL3; Km^r^	This study
pKES3303	*E. coli*–*Kocuria* shuttle vector harboring 1.4 kbp fragment from pKPAL3; Km^r^	This study
pKES3305	*E. coli*–*Kocuria* shuttle vector harboring 3.4 kbp fragment from pKPAL3; Km^r^	This study
pKES3306	*E. coli*–*Kocuria* shuttle vector harboring 2.5 kbp fragment from pKPAL3; Km^r^	This study
pKES3307	*E. coli*–*Kocuria* shuttle vector harboring 1.5 kbp fragment from pKPAL3; Km^r^	This study
pKES3309	*E. coli*–*Kocuria* shuttle vector harboring 1.4 kbp fragment from pKPAL3; Km^r^	This study
pKES3310	*E. coli*–*Kocuria* shuttle vector harboring 1.0 kbp fragment from pKPAL3; Km^r^	This study
pKES3311	*E. coli*–*Kocuria* shuttle vector harboring 0.6 kbp fragment from pKPAL3; Km^r^	This study
pKES3312	*E. coli*–*Kocuria* shuttle vector harboring 0.8 kbp fragment from pKPAL3; Km^r^	This study
pKES3300Δorf1	*E. coli*–*Kocuria* shuttle vector harboring 3.7 kbp fragment from pKPAL3; Km^r^	This study
pKES3300Δorf2	*E. coli*–*Kocuria* shuttle vector harboring 3.4 kbp fragment from pKPAL3; Km^r^	This study
pKES3300Δorf12	*E. coli*–*Kocuria* shuttle vector harboring 2.4 kbp fragment from pKPAL3; Km^r^	This study
pKES3300Δorf3	*E. coli*–*Kocuria* shuttle vector harboring 4.0 kbp fragment from pKPAL3; Km^r^	This study
pKES3300Δorf4	*E. coli*–*Kocuria* shuttle vector harboring 3.5 kbp fragment from pKPAL3; Km^r^	This study
pKITE301	*E. coli*–*Kocuria* shuttle vector harboring 1.0 kbp fragment from pKPAL3; Km^r^	This study
pKITE301P	*Lac* promoter of pKITE301 is replaced by *gapdh* promoter of *K. rhizophila* DC2201	This study
pKITE303	*E. coli*–*Kocuria* shuttle vector harboring 1.0 kbp fragment from pKPAL3; Thio^r^	This study
pKITE103P-LSADH	LSADH expression vector	Toda et al., 2017

### Isolation and Characterization of pKPAL3 from *K. palustris* IPUFS-1

Standard techniques were used for DNA manipulation (Sambrook and Russell, 2001). Cryptic plasmids were extracted from *K. palustris* IPUFS-1 cells using a previously described method (Toda et al., 2017). To obtain pure plasmid samples, extracted plasmids were purified by CsCl density-gradient ultracentrifugation and separated by agarose gel electrophoresis. Plasmids were then extracted from agarose gel and purified using the Wizard SV Gel and PCR Clean-Up System (Promega Corp.). Transposons were inserted into the purified plasmid with the EZ-Tn5TM <R6Kγ*ori*/KAN-2: kanamycin resistant> Insertion Kit (AR Brown, Tokyo, Japan) according to the manufacture’s protocol. Plasmids with transposons inserted were introduced into *E. coli* EC100D *pir*-116 competent cells, and transformed cells were selected on LB agar plates containing 100 μg/mL kanamycin. Selected transformants were cultured in 4 mL of LB liquid medium containing 100 μg/mL kanamycin, and the plasmids were prepared using the Wizard Plus SV Minipreps DNA purification system (Promega Corp.). DNA sequencing was conducted using a 3130 capillary DNA sequencer (Applied Biosystems, Foster City, CA, United States) to determine the nucleotide sequences of the obtained plasmid. The primers used for the determination of the nucleotide sequences are shown in Supplementary Table S1.

### Construction of *E. coli–Kocuria* Shuttle Vectors

**FIGURE 1:**
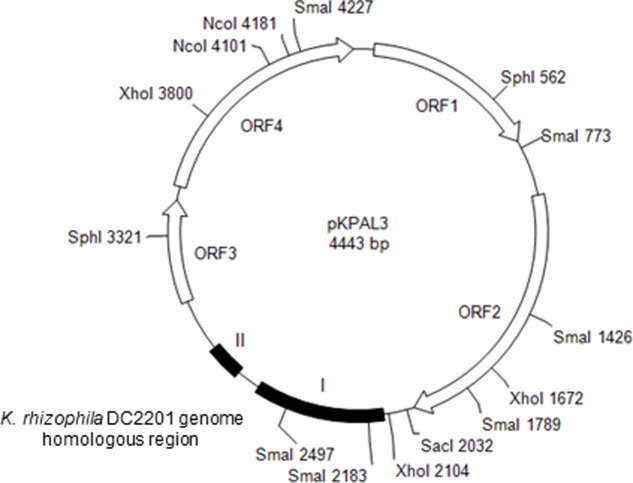
Structure of pKPAL3 from *Kocuria palustris* IPUFS-1. Predicted ORFs are shown as arrows. Black boxes indicate regions of homology with genomic DNA from *K. rhizophila* DC2201.

To determine the minimum region of pKPAL3 required for autonomous replication in *Kocuria* cells, deletion clones were derived from pKPAL3 (**Figure 1**). Various pKPAL3 DNA fragments were obtained by PCR using the primers listed in Supplementary Table S1. The amplified DNA fragments were treated with the restriction endonucleases *Bam*HI and *Sal*I and ligated with pHSG298 that had been amplified by PCR and incubated with *Bgl*II and *Sal*I. Ligated plasmids were then transformed into *E. coli* JM109 and selected on LB agar plates containing 100 μg/mL kanamycin. *K. rhizophila* DC2201 cells were transformed with pKPAL3 derivatives (pKES3300 to pKES3312 and pKES3300Δorf1 to pKES3300Δorf4) according to methods detailed in our previous report (Toda et al., 2017), and transformants were selected on SOB agar (2% [*w/v*] tryptone, 0.5% [*w/v*] yeast extract, 10 mM NaCl, 2.5 mM KCl, 10 mM MgSO_4_, 10 mM MgCl_2_, pH 7.0) containing 400 μg/mL kanamycin.

**FIGURE 2:**
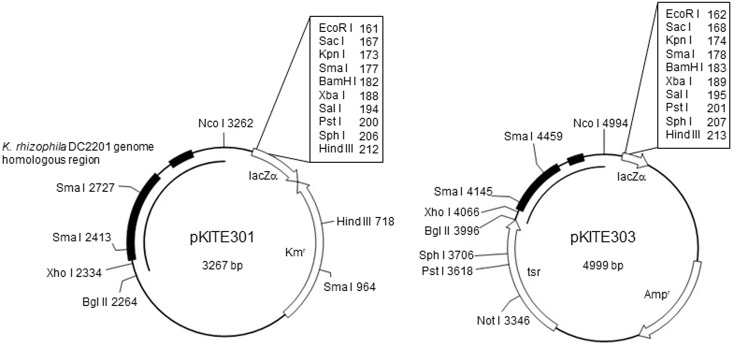
Structure of the *E. coli*–*Kocuria* shuttle vectors derived from pKPAL3. Representative restriction endonuclease sites are shown. Solid arrows indicate the ORFs and their directions of transcription. Black boxes indicate regions of homology with the *K. rhizophila* DC2201 genome. Km^r^, kanamycin resistance gene (*kanR*); Amp^r^, ampicillin resistance gene (*bla*); Thio^r^, thiostrepton resistance gene (*tsr*).

To construct the pKITE301 vector (**Figure 2**), the PCR-amplified fragment of pKPAL3 (amplified using primers pKPAL3Fdel5nco and pKPAL3Rdel2bgl) was incubated with the restriction endonucleases *Nco*I and *Bgl*II and ligated with a portion of pHSG298 that was PCR amplified using primers pHSG298Fbgl and pHSG298Rnco and incubated with the same restriction endonucleases. The ligated plasmid was transformed into *E. coli* JM109 to yield pKITE301. Similarly, the PCR fragment of pKPAL3 described above was ligated with a portion of pUC118 PCR amplified using primer pHSG298Fbgl and pHSG298Rnco and incubated with *Nco*I and *Bgl*II, and it was then transformed into *E. coli* JM109. The obtained plasmid was cut with *Bgl*II and ligated with the thiostrepton resistance gene (*tsr*) amplified by PCR to yield pKITE303. All transformation experiments were performed three times, and transformation efficiencies were expressed as means and standard deviations (SDs).

Plasmid copy number was estimated using real-time qPCR, and plasmid stability in *Kocuria* cells was determined according to the methods described in a previous study (Toda et al., 2017). The primers used for estimating copy number by real-time qPCR are shown in Supplementary Table S1.

### Transformation of Various *Kocuria* Species

Competent *Kocuria* spp. cells were prepared by washing cells with glycerol solution as described previously (Toda et al., 2017). Each microorganism was grown on LB medium supplemented with 1.5% (*w*/*v*) glycine, except *K. polaris* NBRC 103063, to an OD_660_ of 0.7 at 30°C with shaking. The harvested cells were washed twice with sterilized water and resuspended in 10% (*v*/*v*) glycerol with OD_660_ adjusted to 50. Electroporation was conducted with 0.5 μg of the pKITE301 plasmid, and cells were cultured on a selection plate (SOB medium containing 100 or 200 μg/mL kanamycin). All transformation experiments using *Kocuria* spp. were performed three times, and transformation efficiencies were expressed as means and SDs.

### Development of a RhSMO and LSADH Co-expressing Biocatalyst

To develop a *K. rhizophila* DC2201 biocatalyst that expresses both RhSMO and LSADH genes, the two plasmids pKITE301P-RhSMO and pKITE103P-LSADH were constructed as follows. First, to construct a pKITE301P plasmid in which the *lac* promoter was replaced with the *gapdh* promotor from *K. rhizophila* DC2201, an *Nde*I restriction site was introduced into multicloning sites of pKITE301 using a PrimeSTAR Mutagenesis Basal Kit (Takara, Shiga, Japan) according to the manufacture’s protocol to yield pKITE301nde. The promotor sequence of *gapdh* was amplified by PCR using *K. rhizophila* DC2201 genomic DNA as a template and introduced into the pKITE301nde vector using the *Nde*I and *Nco*I restriction sites. The obtained plasmid was designated pKITE301P. The RhSMO gene was amplified by PCR using *Rhodococcus* sp. ST-10 genomic DNA as a template, and the PCR product was inserted into pKITE103P using *Nde*I and *Bam*HI sites. A PCR fragment of the LSADH gene from *Leifsonia* sp. S749 was inserted into pKITE103P (Toda et al., 2017) using the *Nde*I and *Bam*HI restriction sites.

The constructed plasmids were used to transform *K. rhizophila* DC2201 cells, and transformants were grown on DC2201 agar medium (0.5% [*w/v*] glucose, 1% [*w/v*] tryptone, 1% [*w/v*] yeast extract, 0.5% [*w/v*] NaCl, 0.3% [*w/v*] bonito extract, pH 7.0) containing 400 μg/mL kanamycin and 10 μg/mL thiostrepton. The transformants harboring both pKITE301P-RhSMO and pKITE103P-LSADH were selected and used for further experiments.

### Bioproduction of (*S*)-Styrene Oxide in a Bi-phasic System

The *K. rhizophila* DC2201 cells expressing RhSMO and LSADH were grown on 50 mL DC2201 medium containing 400 μg/mL kanamycin and 10 μg/mL thiostrepton at 30°C with shaking. After cultivation, the culture medium (50 mL) was supplemented with 2.5 mL of 1 M MOPS buffer (pH 7.5) to stabilize its pH and 25 mL of DEHP containing 400 mM styrene and an appropriate amount of 2-propanol (5% to 20% [*v*/*v*] in DEHP). Bioconversion was conducted at 30°C with shaking, and an aliquot of the organic layer (500 μL) was collected after centrifugation for 5 min. The collected samples were dried with anhydrous Na_2_SO_4_ and dissolved in ethyl acetate. Then, gas chromatography analysis was performed to determine the concentrations of styrene and (*S*)-styrene oxide in the reaction mixture.

### Gas Chromatography

Products in the reaction mixture were analyzed using an HP 6890 GC system (Agilent Technologies, Tokyo, Japan) equipped with a Chrompack CP-cyclodextrin-β-2,3,6-M19 chiral capillary column (0.25 mm × 25 m, 0.25 μm film; Valian, Palo Alto, CA, United States). Helium gas was used as the carrier at 15 psi, and the split ratio was 50. Both the injector and detector temperatures were 250°C. Measurement of the product was performed isothermally (at a column temperature of 105°C) with a flame ionization detector. The retention times of styrene, (*R*)-styrene oxide, and (*S*)-styrene oxide were 2.90, 7.50, and 7.69 min, respectively.

### Nucleotide Sequence Accession Numbers

The determined nucleotide sequences of pKPAL3 from *K. palustris* IPUFS-1, pKITE301, pKITE303, and pKITE301P were registered with the DNA Data Bank Japan (DDBJ) under accession numbers LC317093, LC317094, LC317095, and LC317096, respectively. The nomenclatures of the constructed vectors should be standardized in the future, according to the ‘Standard European Vector Architecture’ database (SEVA-DB) for a coherent platform of molecular tools that are subject to a concise, and standardized format and nomenclature (Silva-Rocha et al., 2013).

## Results

### Isolation and Characterization of pKPAL3

We isolated the novel cryptic plasmid pKPAL3 from *K. palustris* IPUFS-1 by using a transposon insertion kit in a manner similar to that described in a previous report (Toda et al., 2017). The isolated plasmid was designated pKPAL3, and its whole nucleotide sequence was determined. pKPAL3 consisted of 4,443 bp with a GC content of 63.5%, and it possessed four estimated ORFs (**Figure 1**). BLAST searches indicated that ORF1, ORF2, and ORF3 showed approximately 40–50% amino acid sequence identity with hypothetical proteins from other organisms, but their physiological functions remain unclear (**Table 2**). ORF4 exhibited an amino acid sequence identity of 48.8% with bacterial integrase/recombinase XerD from *Nocardiaceae bacterium* Broad-1. However, we were unable to identify genes encoding replication proteins in pKPAL3. There were two nucleotide sequence regions that showed significant identities with untranslated genomic regions of *K. rhizophila* DC2201 (black boxes in **Figure 1**). Region I consisted of 504 nucleotides and showed 76% identity with the *K. rhizophila* DC2201 genome (positions 61704–62167). Region II consisted of 134 nucleotides, and it showed 77–84% identities with three homologous regions in the *K. rhizophila* DC2201 genome (positions 790542–790676, 799495–799629, and 1921252–1921379). Furthermore, microorganisms belonging to *Micrococcaceae* such as *K*. *turfanensis* and *Micrococcus luteus* possessed chromosomal or endogenous plasmid sequences that were homologous to portions of region II (**Table 2**).

**Table 2:** BLAST search analysis of putative ORFs of pKPAL3 from *Kocuria palustris* IPUFS-1.

ORF	Position	BLAST search	Identity (%)	Accession no.
ORF1	63-743	Hypothetical protein from *Streptococcus pneumoniae* PCS70012	53.5	ELU61146
ORF2	966-2003	Hypothetical protein from *Corynebacterium epidermidicanis*	50.0	AKK03111
ORF3	3061-3459	Hypothetical protein from *Cellulomonas cellasea* DSM 20118	41.7	KGM01696
ORF4	3512-4426	Putative integrase/recombinase XerD from *Nocardioidaceae bacterium* Broad-1	48.8	EGD42648

**Region**	**Position**	**Homologous sequence (positions)**	**Identity (%)**	**Accession no.**

Region I	2123-2626	*K. rhizophila* DC2201, complete genome (61704 to 62167)	76	AP009152.1
Region II	2721-2854	*K. rhizophila* DC2201, complete genome (799495 to 799629; 790542 to 790676; 1921252 to 1921379)	77–84	AP009152.1
		*K. turfanensis* strain HO-9042 genome (2647231 to 2647361; 3417000 to 3417130; 380434 to 380564; 1459868 to 1459991; 3357701 to 3357842; 2547137 to 2547259)	80–81	CP014480.1
		*K. turfanensis* strain HO-9042 plasmid unnamed1 (103019 to 103143; 1885 to 2014; 33792 to 33917; 109507 to 109631)	79–80	CP014481.1
		*K. turfanensis* strain HO-9042 plasmid unnamed4 (28066 to 28195)	79	CP014483.1
		*Micrococcus luteus* NCTC2665 complete genome	77–82	CP001628.1
		(2439192 to 2439324; 344175 to 344304; 2304461 to 2304592; 577570 to 577702; 1526931 to 1527030; 1558046 to 1558175)		
		*Micrococcus* sp. A7 plasmid pLMA7 (65195 to 65324; 79792 to 79924)	78–81	KJ599675.1
		*Micrococcus* sp. 28 plasmid pSD10 (12672 to 12802; 16904 to 17034)	80	AY034092.1
		*Micrococcus* sp. A1 plasmid pLMA1 (3973 to 4104)	78	LK056645.1
		*Micrococcus* sp. V7 plasmid pLMV7 (61981 to 62111)	79	KF577591.1

### Minimum Sequence Region Required for Autonomous Replication in *K. rhizophila* DC2201

**FIGURE 3:**
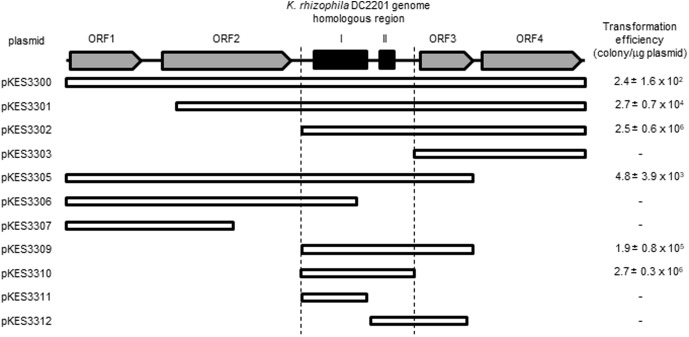
Determination of the minimum region for self-replication of pKPAL3 derivatives in *K. rhizophila* DC2201 and their transformation efficiencies. Numbers indicate the transformation efficiency of the plasmids that are capable of self-replication in *K. rhizophila* cells.

We determined the minimum nucleotide sequence required for self-replication of pKPAL3 in *K. rhizophila* DC2201 through the construction of pKPAL3 deletion plasmids. Positive transformant cells were obtained that had plasmids containing both regions I and II; however, predicted ORFs encoding hypothetical proteins or the predicted recombinase (XerD) were unnecessary for autonomous replication (**Figure 3**).

### Transformation Efficiency

**FIGURE 4:**
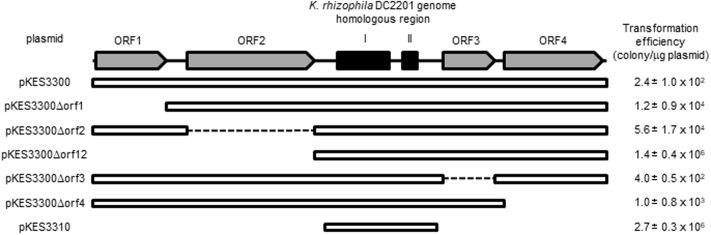
Transformation efficiency of ORF-deleted variants derived from pKES3300. Numbers indicate the transformation efficiency of the plasmids that are capable of self-replication in *K. rhizophila* cells.

As shown in **Figure 3**, there were significant differences in transformation efficiencies for *K. rhizophila* DC2201 among the various deletion plasmids. For example, pKES3310 consisted of the minimum region for replication and exhibited a transformation efficiency that was four orders of magnitude higher than that of pKES3300 possessing the entire pKPAL3 plasmid. To investigate the effect of each ORF on transformation efficiency, we constructed deletion plasmids that lacked each of the predicted ORFs and examined the transformation efficiency of host *K. rhizophila* DC2201 cells with each deletion plasmid (**Figure 4**). ORF1 and ORF2 were revealed to have a negative effect on transformation efficiency. As expected, the elimination of these ORFs from the plasmid increased the transformation efficiency of constructed plasmids by more than 100-fold compared with that of pKES3300. On the other hand, deletion of ORF3 and/or ORF4 showed much less effect on the transformation efficiency relative to the deletion of ORF1 or ORF2.

### Plasmid Copy Number and Segregation Stability

To estimate the copy number of pKITE301 in *K. rhizophila* DC2201, qPCR analysis was conducted on transformed *K. rhizophila* DC2201 using an approach that was similar to that described in a previous report that targeted *tpi*, *pgk*, and *eno* genes in chromosomal DNA and *kanR* in plasmid DNA (Toda et al., 2017). The copy number of pKITE301 was calculated to be 20 per chromosome by the ratio of the mean copy number of *kanR* gene to those of *tpi*, *pgk*, and *eno*.

**FIGURE 5:**
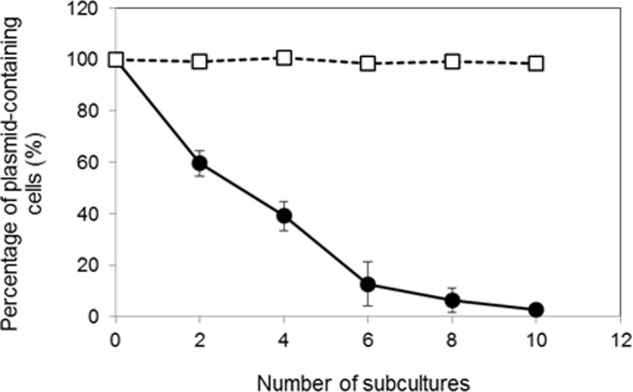
Comparison of segregation stability of pKITE301 and pKITE101 in *K. rhizophila* DC2201 cells. The percentage of transformant cells possessing shuttle vectors after each subculture passage without selective antibiotics is shown: 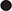, pKITE301; 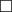, pKITE101 (data from Toda et al., 2017). The error bars indicate standard deviations (SDs) of three measurements.

We examined the segregation stability of pKITE301, pKES3300 and the pKES3300 deletion plasmids (pKES3300Δorf1 to pKES3300Δorf4; **Figure 4**) in *K. rhizophila* DC2201 transformant cells. As shown in **Figure 5**, pKITE301 exhibited much lower segregation stability than pKITE101, and the number of antibiotic-resistant cells harboring plasmids decreased to about 2% of that of the original population after 10 subculture passages. Furthermore, a substantial decrease of segregation stability was observed only for pKES3300Δorf4 and pKITE101, while other deletion plasmids were stably maintained in *K. rhizophila* DC2201 cells (Supplementary Figure S1).

### Compatibility of pKITE301 and pKITE103 in *Kocuria* Cells

To confirm the compatibility of the novel shuttle vector pKITE301 (Km^r^), the vector was transformed into *K. rhizophila* DC2201 cells harboring pKITE103 (Thio^r^), and transformant cells that demonstrated resistance to both kanamycin and thiostrepton were selected. *K. rhizophila* cells harboring both plasmids were then confirmed by agarose gel electrophoresis analysis. Both plasmids were compatible with each other, and they were able to simultaneously replicate in *K. rhizophila* transformant cells in the presence of antibiotics. We also confirmed that pKITE301 and pKITE103 derivatives are able to coexist with the *Kocuria–E. coli* shuttle vector pCK-PSD1 developed by Matsumura et al. (2012) in *K. rhizophila*, indicating they are compatible with each other (data not shown).

### Transformation of *Kocuria* Species with pKITE301

The self-replication of the constructed shuttle vectors was tested in other *Kocuria* species (**Table 3**). pKITE301 showed a broad range of compatibility with various *Kocuria* species, and the transformation efficiencies for these microorganisms were two to four orders of magnitude higher than those for pKITE101 except for *K. rhizophila* NBRC 16319. However, no successful transformant was observed for *K. kristinae* NBRC 15354 and *K. polaris* NBRC 103063. We also attempted transformation of *M. luteus* NBRC3333 cells using pKITE301, but no transformant was observed (data not shown).

**Table 3:** Transformation efficiencies of pKITE301 and pKITE101 for *Kocuria* species.

Strain	Transformation efficiency of pKITE301 (cfu/μg DNA)	Transformation efficiency of pKITE101 (cfu/μg DNA)^a^
*K. rhizophila* DC2201	2.7 ± 0.3 × 10^6^	5.3 × 10^2^
*K. kristinae* NBRC 15354	ND^b^	ND
*K. varians* NBRC 15358	4.7 ± 1.1 × 10^5^	2.8 × 10^3^
*K. palustris* NBRC 16318	1.1 ± 0.2 × 10^4^	1.5 × 10^2^
*K. rhizophila* NBRC 16319	8 ± 1.4 × 10	5 × 10^2^
*K. polaris* NBRC 103063	ND	ND
*K. flava* NBRC 107626	2.5 ± 1.4 × 10^6^	ND
*K. turfanensis* NBRC 107627	3.1 ± 0.3 × 10^5^	1 × 10^2^

^a^*Data from Toda et al. (2017).*

^b^*ND, not detected*.

### Bioproduction of (*S*)-Styrene Oxide Using a *K. rhizophila* RhSMO-LSADH Co-expression Biocatalyst

To develop biocatalysis systems for producing enantiopure epoxides, the RhSMO-coding gene was heterologously expressed in *K. rhizophila* DC2201 cells under the control of a *gapdh* promotor using pKITE301P. LSADH, an alcohol dehydrogenase from *Leifsonia* sp. S749, was also expressed in *K. rhizophila* cells using pKITE103P for regenerating NADH by using 2-propanol as a hydrogen donor. Bioconversion of styrene to (*S*)-styrene oxide was conducted in the organic solvent–water biphasic system because the produced epoxide is unstable under aqueous conditions. **Table 4** shows that the biocatalyst expressing both RhSMO and LSADH genes successfully produced (*S*)-styrene oxide in the presence of 2-propanol. Under the same conditions, a negligible amount of product was obtained by *Kocuria* cells possessing pKITE301P-RhSMO/pKITE103P. The control biocatalyst harboring pKITE301P/pKITE103P-LSADH showed no production of styrene oxide.

**Table 4:** Comparison of styrene oxide production by *K. rhizophila* DC2201 biocatalysts.

Plasmid	Hydrogen source^a^	Gene	Styrene oxide (mM)^b^
pKITE301P-RhSMO/pKITE103P	2-propanol	RhSMO	1.1 ± 0.8
pKITE301P-RhSMO/pKITE103P-LSADH	2-propanol	RhSMO, LSADH	47.0 ± 2.7
pKITE301P/pKITE103P-LSADH	2-propanol	LSADH	0

^a^*Reactions were performed in the presence of 5% (*w*/*v*) 2-propanol in the organic solvent phase with 100 mM styrene. ^b^Product concentration in the organic phase*.

**FIGURE 6:**
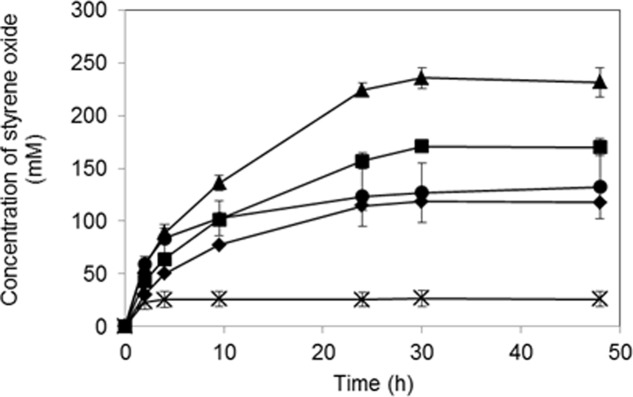
Bioproduction of (*S*)-styrene oxide using *K. rhizophila* DC2201 biocatalysts co-expressing the RhSMO and LSADH genes. Symbols indicate 2-propanol concentrations in the bis-(2-ethylhexyl) phthalate (DEHP) organic solvent phase: 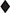, 5% (*v*/*v*); 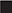, 10% (*v*/*v*); 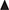, 15% (*v*/*v*); 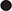, 20% (*v*/*v*);×, 25% (*v*/*v*). The errors bars indicate SDs of three measurements.

To optimize the bioconversion, the effect of 2-propanol concentration in the organic solvent was investigated. As shown in **Figure 6**, the product concentration generally increased in accordance with the increased 2-propanol concentration, and the highest production level of (*S*)-styrene oxide was obtained in the presence of 15% (*v*/*v*) 2-propanol in the organic solvent. Under this condition, the product concentration reached 235 mM in a 30-h reaction. However, styrene oxide production decreased at a 2-propanol concentration of 20% (*v/v*), and a low yield of (*S*)-styrene oxide was obtained at a 25% (*v/v*) 2-propanol concentration.

## Discussion

Recently, we demonstrated that *K. rhizophila* DC2201 was a suitable host cell strain for producing aliphatic epoxyalkanes because they have robust cell structure and organic solvent tolerance (Toda et al., 2015). Developing genetic tools for *Kocuria* spp. is important for increasing the availability of this microorganism for industrial use to produce various organic compounds.

In our previous study, we isolated the two cryptic plasmids pKPAL1 and pKPAL2 from *K. palustris* IPUFS-1 using the transposon-plasmid rescue method (Toda et al., 2017). Unfortunately, we were unable to generate shuttle vectors from pKPAL2. In that study, other plasmids were detected in the extracted plasmid sample. Thus, *K. palustris* IPUFS-1 was assumed to have more than three cryptic plasmids in their cells. In order to construct a biocatalyst, a combination of compatible plasmid vectors expressing multiple genes in one microorganism cell is often required. In this study, we successfully identified the cryptic plasmid pKPAL3 from *K. palustris* IPUFS-1 and constructed a novel *E. coli*–*Kocuria* shuttle vector that is compatible with the pKITE101 plasmid previously constructed.

pKPAL3 possesses four putative ORFs, which showed high sequence identity with hypothetical proteins from Gram-positive bacteria, although the physiological roles of all ORFs remain unclear except for that of ORF4 (**Table 2**). The amino acids sequence of ORF4 showed high sequence identity with the putative recombinase/integrase XerD from *N. bacterium* Broad-1. Blakely et al. (1993) reported that the XerC/D recombinase system is involved in the stable inheritance of the ColE-1-related circular plasmid, and it also maintains a portion of chromosomal DNA from *E. coli* (Blakely and Sherratt, 1994). Furthermore, the *resU* gene, which is a member of the XerD recombinase gene family in the cryptic plasmid pUE10 from *Deinococcus radiodurans*, is involved in the stable replication and inheritance of pUE10 derivatives, and Δ*resU* derivatives decreased in segregation stability in *D. radiodurans* cells (Meima and Lidstrom, 2000). These findings suggest that ORF4 may be involved in stabilizing segregation and inheritance of pKPAL3 in *Kocuria* cells. Indeed, segregation stability significantly decreased only in pKES3300Δorf4 compared with original pKES3300 and other ORF-deleted derivatives as shown in Supplementary Figure S1. This result supported our speculation.

Notably, pKPAL3 contained two nucleotide sequence regions (regions I and II; **Figure 1**) that showed significant sequence identities with genomic DNA from *K. rhizophila* DC2201. At least one and three conserved nucleotide sequences corresponding to regions I and II of pKPAL3 were confirmed in *K. rhizophila* DC2201 genomic DNA, respectively. These nucleotide sequences are located upstream of the IS 1380 family transposase (region I) and the IS481 family transposase (region II), and neither codes for a polypeptide. Additionally, several *Kocuria* and *Micrococcus* species, including *K. turfanensis* and *M. luteus*, have sequences that are homologous to regions I and II in their chromosomal and plasmid sequences (**Table 2**). Most of these sequences are located upstream of transposases as observed in *K. rhizophila* DC2201. These findings suggested that such nucleotide sequences have been horizontally transferred from the same origin via the transposase gene.

To determine the minimum sequence region required for autonomous replication of pKPAL3 in *Kocuria* cells, deletion plasmids of pKPAL3 were constructed and used to transform a series of *K. rhizophila* DC2201 cells. **Figure 3** shows that no predicted ORFs are required for autonomous replication, whereas regions I and II are essential for self-replication of pKPAL3 in *Kocuria* cells. Furthermore, we found that transformation efficiency varied among plasmid derivatives. Therefore, we investigated the effect of each ORF on transformation frequency using a deletion series. The results shown in **Figure 4** revealed that the deletion of each ORF except ORF3 increased transformation efficiency of pKPAL3 plasmids. Particularly, the deletion of ORF1 and/or ORF2 substantially increased transformation efficiency, whereas the effect of ORF4 deletion was less than that of ORF1 or ORF2 deletion. Maier et al. (2004) also reported that a deletion derivative of an *E. coli*–*Francisella* shuttle vector lacking several ORFs exhibited higher transformation frequency than did the native plasmid. It is generally known that transformation efficiency is affected by modifications of plasmids by methylation in host cells and the configuration of plasmid DNA with respect to oligomerization and catenation (Ehrlich, 1977; Gryczan et al., 1978; Mottes et al., 1979; Canosi et al., 1981). Therefore, we deduced that differences in transformation efficiency of pKPAL3 derivatives were a consequence of the configurations or modifications caused by the deletion of ORFs in each plasmid, although the specific mechanism of this effect remains unclear.

To estimate the copy number of pKITE301 in culture, qPCR was performed using whole DNA extracted from *K. rhizophila* DC2201 harboring pKITE301 as a DNA template. In culture, pKITE301 showed lower copy number than pKITE101 (Toda et al., 2017), and it was calculated to be 20 per chromosome. In comparison with pKITE101, pKITE301 exhibited lower segregation stability and disappeared during culture in the absence of antibiotics (**Figure 5**). Several reports have indicated that theta-replicating plasmids are stably maintained in Gram-positive bacteria cultures (Kiewiet et al., 1993; Zhang et al., 1994; Seegers et al., 1995; Lee and O’Sullivan, 2006), and pKITE101 is also stably maintained in *Kocuria* cells (Toda et al., 2017). In contrast, pKITE301 exhibited poor stability in *Kocuria* cells in the absence of antibiotics. These results suggest that the replicating mechanism of pKPAL3 is different from that of the highly stable theta-type plasmid. As described earlier, the low stability of pKITE301 may be restored by the addition of ORF4 into pKITE301, although further experiments are necessary to confirm this conclusion.

The transformation of various *Kocuria* species by the constructed shuttle vectors was tested. As shown in **Table 3**, several *Kocuria* species, including *K. rhizophila* NBRC 16319, *K. varians* NBRC 15358, *K. palustris* NBRC 16318, *K. flava* NBRC 107626, and *K. turfanensis* NBRC 107627, were successfully transformed with pKITE301 at transformation efficiencies of 8 × 10 to 2.5 × 10^6^ cfu/mg DNA; however, no transformants were observed for *K. kristinae* NBRC 15354 or *K. polaris* NBRC 103063. Further optimization of conditions to prepare competent cells may be needed for these species and/or strains. Additionally, it is generally known that most bacteria have restriction modification (RM) systems that prevent the entry of foreign DNA into the host cell (Wilson and Murray, 1991). Two host cells, *K. kristinae* and *K. polaris*, are likely to have eliminated pKITE301 via their RM systems, or these strains were not competent under the general cell-transformation procedure; either of these conclusions requires confirmation. In most cases, the transformation efficiencies of pKITE301 for *Kocuria* species exceeded those of pKITE101, except for *K. rhizophila* NBRC 16319. These results indicate that the constructed shuttle vectors can be used for genetic modification of various *Kocuria* species.

To confirm the utility of constructed plasmids for heterologous gene expression, we constructed the RhSMO-expression plasmid pKITE301P-RhSMO and the LSADH-expression plasmid pKITE103P-LSADH (Toda et al., 2017), and both plasmids were simultaneously transformed into *K. rhizophila* DC2201 cells. Under the control of the *gapdh* promotor from *K. rhizophila*, both RhSMO and LSADH genes were successfully expressed in transformed cells (data not shown). Using *K. rhizophila* cells (i.e., pKITE301P-RhSMO and pKITE103P-LSADH) as a biocatalyst, we conducted bioconversion of styrene to (*S*)-styrene oxide in an organic solvent–water biphasic reaction system. The biocatalyst harboring both RhSMO and LSADH successfully converted styrene to (*S*)-styrene oxide using 2-propanol as a hydrogen donor, whereas negligible amounts of product were obtained in the absence of 2-propanol (**Table 4**). Furthermore, no product was observed when a biocatalyst possessing only LSADH was used. These results indicate that both RhSMO and LSADH were successfully expressed and that LSADH functioned as a NADH regenerator in *Kocuria* cells. These results suggest that these shuttle vectors are more broadly applicable for constructing multi-enzymatic cascade systems for producing various compounds in *Kocuria* cells.

The concentration of 2-propanol (the hydrogen donor for NADH regeneration by LSADH) in the organic solvent phase is an important factor for producing styrene oxide because it affects not only the solubility of the substrate in the aqueous phase but also the inactivation of the biocatalyst. When 15% (*v/v*) 2-propanol was added to the organic solvent (i.e., DEHP) phase, the highest product concentration (235 mM) was achieved after a 30-h reaction (**Figure 6**). On the other hand, styrene oxide production significantly decreased at a 2-propanol concentration of 20% (*v/v*), and the lowest production level was observed for 25% (*v/v*) 2-propanol. Thus, *K. rhizophila* biocatalysts possessing the RhSMO–LSADH enzyme system exhibited the maximum epoxidation rate in the presence of 15% (*v/v*) 2-propanol and showed resistance to 20% (*v/v*) 2-propanol. In corresponding *E. coli* recombinant biocatalyst systems, the optimum 2-propanol concentration was 6% (*v/v*), and the reaction was greatly inhibited at concentrations exceeding 10% (*v/v*), although the conditions were slightly different (Toda et al., 2012a). Thus, the *K. rhizophila* biocatalyst system had higher 2-propanol resistance than the corresponding *E. coli* system and achieved higher yields of (*S*)-styrene oxide (235 mM) than that of the *E. coli* biocatalyst system (150 mM).

We expect that the shuttle vectors developed in this study will be a powerful tool for modification of some *Kocuria* spp. and developing *K. rhizophila* biocatalysis systems to produce various useful water-insoluble compounds via organic synthesis.

## Author Contributions

HT performed the construction of the plasmid vectors from pKPAL3 and characterization of these plasmid vectors for *Kocuria* sp. and wrote those sections. NI planned the experimental design and wrote some parts of the manuscript.

## Conflict of Interest Statement

The authors declare that the research was conducted in the absence of any commercial or financial relationships that could be construed as a potential conflict of interest.
