# Supplementary figures and images for: Development of a Novel Escherichia coli–Kocuria Shuttle Vector Using the Cryptic pKPAL3 Plasmid from K. palustris IPUFS-1 and Its Utilization in Producing Enantiopure (S)-Styrene Oxide
Source: Front Microbiol. 2017 Nov 27;8:2313. doi: 10.3389/fmicb.2017.02313 (PMC5711781; doi:10.3389/fmicb.2017.02313)

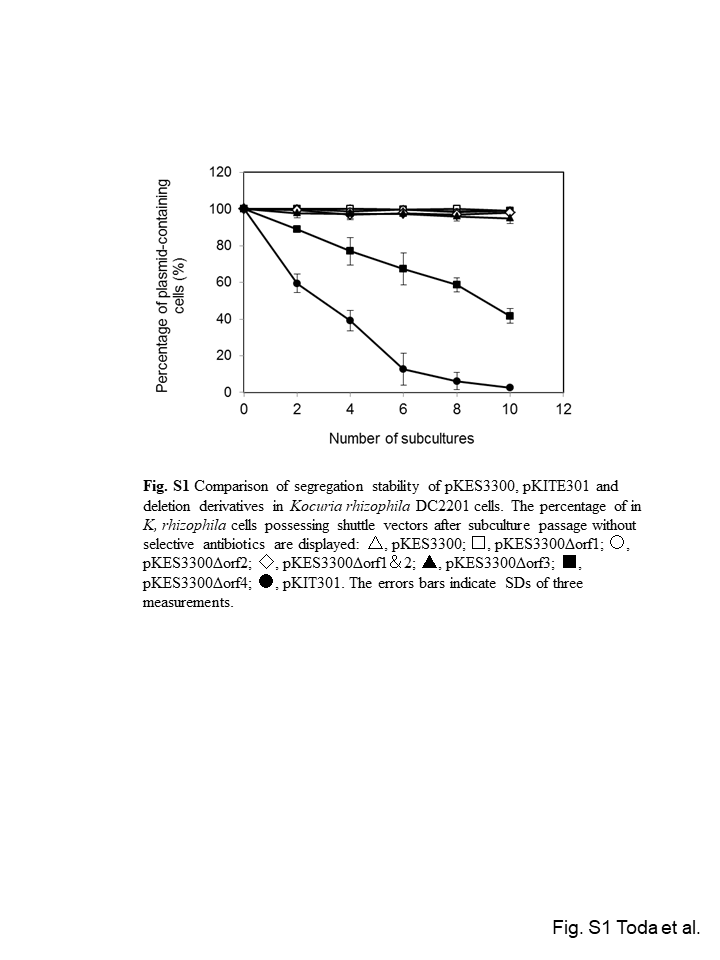

Supplement: Supplementary file 2 [file Image_1.TIF]
